# Whole-transcriptome profiles of *Chrysanthemum seticuspe* improve genome annotation and shed new light on mRNA–miRNA–lncRNA networks in ray florets and disc florets

**DOI:** 10.1186/s12870-022-03889-y

**Published:** 2022-11-05

**Authors:** Daojin Sun, Jing Zhang, Jun He, Zhiqiang Geng, Song Li, Jiali Zhang, Peiling Li, Lingling Zhang, Zhenxing Wang, Likai Wang, Fadi Chen, Aiping Song

**Affiliations:** 1 State Key Laboratory of Crop Genetics and Germplasm Enhancement, Key Laboratory of Landscaping, Ministry of Agriculture and Rural Affairs, College of Horticulture, Nanjing Agricultural University, Nanjing, 210095 China; 2 Henan Key Laboratory of Tea Comprehensive utilization in South Henan, Xinyang Agriculture and Forestry University, Xinyang, 464000 China

**Keywords:** *Chrysanthemum seticuspe*, Genome annotation, ceRNA, lncNAT, Flower development

## Abstract

**Background:**

*Chrysanthemum seticuspe* has emerged as a model plant species of cultivated chrysanthemums, especially for studies involving diploid and self-compatible pure lines (Gojo-0). Its genome was sequenced and assembled into chromosomes. However, the genome annotation of *C. seticuspe* still needs to be improved to elucidate the complex regulatory networks in this species.

**Results:**

In addition to the 74,259 mRNAs annotated in the *C. seticuspe* genome, we identified 18,265 novel mRNAs, 51,425 novel lncRNAs, 501 novel miRNAs and 22,065 novel siRNAs. Two C-class genes and *YABBY* family genes were highly expressed in disc florets, while B-class genes were highly expressed in ray florets. A WGCNA was performed to identify the hub lncRNAs and mRNAs in ray floret- and disc floret-specific modules, and *CDM19*, *BBX22*, *HTH*, *HSP70* and several lncRNAs were identified. ceRNA and lncNAT networks related to flower development were also constructed, and we found a latent functional lncNAT–mRNA combination, LXLOC_026470 and *MIF2*.

**Conclusions:**

The annotations of mRNAs, lncRNAs and small RNAs in the *C. seticuspe* genome have been improved. The expression profiles of flower development-related genes, ceRNA networks and lncNAT networks were identified, laying a foundation for elucidating the regulatory mechanisms underlying disc floret and ray floret formation.

**Supplementary Information:**

The online version contains supplementary material available at 10.1186/s12870-022-03889-y.

## Background

Comprising approximately 25,000 species, Asteraceae is the largest family of flowering plants, with members occurring across all continents except Antarctica [1]. Plants of Asteraceae are characterized by complex inflorescences (capitulum), which include both disc florets in the center and ray florets outside. There are only pistil and zygomorphic petals on ray florets, while there are developed pistil, stamen and actinomorphic petals on disc florets [2]. Chrysanthemums (*Chrysanthemum morifolium* Ramat.) are among the most important cut flowers in the world, are traditional flowers in China, and have high economic value [3]. Due to both polyploidy and hybridization within the genus *Chrysanthemum*, it is difficult to understand the evolution of these species and their underlying molecular basis [4]. *Chrysanthemum seticuspe*, a model plant species of cultivated chrysanthemum plants, has been used for studies involving diploid and self-compatible pure lines (Gojo-0) [5]. The whole genome of Gojo-0 has been sequenced and assembled at the chromosome level [6]. However, organ-specific whole transcriptomes, including mRNAs, lncRNAs and miRNAs, have not been sequenced. To better elucidate the regulatory mechanism of ray florets and disc florets, these molecular networks should be studied.

The development of floral organs is determined by ABCE genes, which regulate floral morphogenesis at various levels together with elaborate networks [7]. The expression levels of ABCE genes determine the specifying organ identity, including that of petals, pistils, sepals and stamens. Suppression of the C-class gene *CAG* results in the conversion of the pistils and stamens into corolla-like tissues in florets of *C. morifolium* [8]. Furthermore, transcription factors other than ABCE genes were also reported to be related to the morphogenesis of florets in the capitulum. Dorsal identity is closely related to the TCP family transcription factor CYCLOIDEA (CYC) [9]. Overexpression of *GhCYC5* increases the flower density in the capitulum of *Gerbera hybrida* [10]. Moreover, knockdown of *CYC2g* in *Chrysanthemum lavandulifolium* promotes the gradual transition from ray florets into disc florets [11]. CUP SHAPED COTYLEDON (CUC) transcription factors were found to limit cell growth and create a creased shape in the boundaries [12]. Additionally, the *YABBY* gene family has been found to specify abaxial cell fate, and mutants of this family showed that they can act in both distinct and redundant manners [13]. However, studies of flower development genes in the genera *Chrysanthemum* and *Gerbera* are still not sufficient to elucidate the formation mechanism of ray florets and disc florets.

Noncoding RNAs (ncRNAs) have been found to be involved in various biological processes in both animals and plants [14]. Including small RNAs and long noncoding RNAs (lncRNAs), ncRNAs participate in almost all biological processes by interacting with coding genes at both the transcriptional and posttranscriptional levels in plants [15]. MicroRNAs (miRNAs) can trigger posttranscriptional repression by targeting mRNA through high complementarity [16]. In tomato, sly-miR160a regulates blade outgrowth, leaf and leaflet initiation and floral organ development by targeting *SlARF10A* and adjusting auxin-mediated development [17]. lncRNAs are RNAs with a length of more than 200 nt and no protein-coding capability; lncRNAs include long intergenic noncoding RNAs (lincRNAs) and long noncoding natural antisense transcripts (lncNATs), which are denoted according to their position relative to genes [18]. lncRNAs regulate biological mechanisms of gene expression, including chromatin remodelling, modulation of alternative splicing, fine tuning of miRNA activity, and the control of mRNA translation or accumulation [19]. A lncNAT (*TWISTED LEAF*) has been shown to influence leaf blade flattening in rice through its regulation of the expression of *OsMYB60* [20]. Another lncRNA (*MISSEN*) in rice binds to a helicase family protein (HeFP), which ultimately damages the protein complex of HeFP and tubulin during endosperm nuclear division, resulting in abnormal cytoskeletal polymerization [21]. LncRNAs can also act as competing endogenous RNAs (ceRNAs) to modulate gene expression by mimicking miRNAs. Both *lncRNA23468* and *NBS-LRR* genes are targeted by miR482b in tomato, and the downregulation of *lncRNA23468* leads to the downregulation of *NBS-LRR* genes [22]. Although lncRNAs play an important role in plant development, their biological mechanisms in flower morphogenesis remain unclear, which prompted us to explore their expression profiles in flowers.

**Fig. 1:**
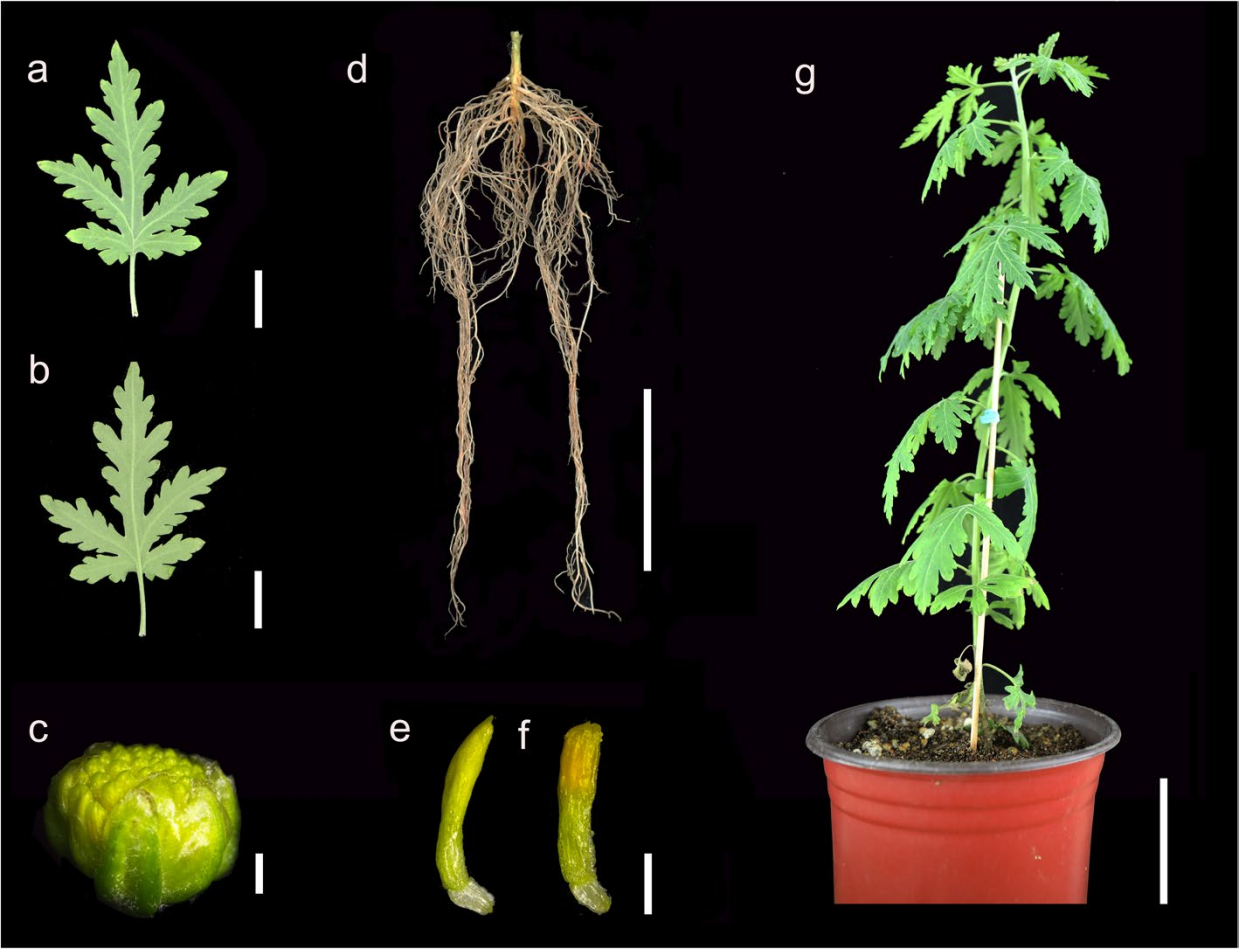
Morphology of five organs sampled from the seedlings of Gojo-0. (a-b) The adaxial surface and abaxial surface of a leaf. Bar, 1 cm. (c) A flower bud at the sampling stage. This stage is the final stage of corolla primordia differentiation according to Wen’s study [25]. Bar, 1 mm. (d) Root. Bar, 5 cm. (e-f) Single ray floret and disc floret of a head-like inflorescence. Bar, 1 mm. (g) Intact seedling. Bar, 5 cm

Whole-transcriptome sequencing is useful for constructing mRNA–miRNA–lncRNA regulatory networks and has been used in *Populus* and *Liriodendron chinense* [23, 24]. In this study, we sequenced whole transcriptomes of mRNAs, lncRNAs and small RNAs in five different organs (leaves, stems, roots, disc florets and ray florets) of *C. seticuspe*, which are shown in Fig. 1. These transcriptomes improved the annotation of the genome and showed overall gene expression profiles. To identify networks related to flower development genes in *C. seticuspe*, latent mRNA–lncRNA-miRNA regulatory networks were also discovered with weighted gene correlation network analysis (WGCNA), positional relationships in the genome and prediction of targeting miRNAs. In summary, our study will contribute to our understanding of the mechanism of lncRNAs in capitulum flower morphogenesis.

## Results

### Transcriptome sequencing

The Q20 values, percentage of clean data and total mapping percentage of the lncRNA, mRNA and small RNA library sequences of each sample are shown in Table 1 and Table 2. The mean Q20 value of the lncRNA and mRNA libraries was more than 97% and that of the small RNA library was more than 98%. The percentage of clean reads in the lncRNA and mRNA libraries was more than 91%, and the percentage of clean tags in the small RNA library was more than 92%. The total mapping percentage of the lncRNA and mRNA libraries was more than 77% and that of the small RNA library was more than 89%. After filtration, a total of 1,844,502,354 clean reads were generated for the lncRNA and mRNA libraries, and a total of 418,586,690 clean tags were generated for the small RNA libraries.

**Table 1:** Results of the lncRNA–mRNA library sequencing of five organs

Sample	Total Raw Reads	Total Clean Reads	Q20 of Fq1	Q20 of Fq2	Clean Read Percentage	Total Mapping Percentage
**disc1**	130,669,850	125,529,528	97.56%	98.39%	96.07%	86.59%
**disc2**	116,002,232	111,871,100	97.48%	98.57%	96.44%	87.05%
**disc3**	110,960,416	106,786,472	97.48%	98.45%	96.24%	86.75%
**leaf1**	131,871,738	126,250,264	98.08%	98.74%	95.74%	78.75%
**leaf2**	132,432,902	127,174,762	97.32%	98.49%	96.03%	80.96%
**leaf3**	132,432,902	126,760,240	97.27%	98.60%	95.72%	79.91%
**ray1**	137,430,370	125,847,802	97.45%	98.61%	91.57%	80.50%
**ray2**	132,432,902	127,349,076	97.41%	98.54%	96.16%	85.50%
**ray3**	118,014,778	113,746,826	97.40%	98.46%	96.38%	86.04%
**root1**	131,871,738	128,082,958	98.01%	98.51%	97.13%	77.50%
**root2**	132,245,604	128,049,146	97.15%	98.59%	96.83%	79.63%
**root3**	129,410,340	125,491,270	97.39%	98.72%	96.97%	78.61%
**stem1**	129,383,592	126,023,890	98.14%	98.66%	97.40%	81.85%
**stem2**	132,432,902	128,157,982	97.68%	98.61%	96.77%	84.14%
**stem3**	121,456,166	117,381,038	97.27%	98.59%	96.65%	84.03%

Note: Fq1 and Fq2 represent fastq1 and fastq2, respectively, which were generated by paired-end sequencing of transcriptomes

**Table 2:** Results of the small RNA library sequencing of five organs

Sample	Raw tag count	Clean tag count	Q20 of clean tags	Percentage of clean tags	Total mapping percentage
**disc1**	28,268,298	27,383,783	99%	96.87%	96.29%
**disc2**	29,766,389	28,193,618	98.80%	94.72%	95.73%
**disc3**	28,203,774	27,179,853	99%	96.37%	96.06%
**leaf1**	29,151,442	27,898,713	98.80%	95.70%	96.33%
**leaf2**	29,995,008	28,446,922	98.70%	94.84%	96.44%
**leaf3**	29,586,790	28,040,952	98.80%	94.78%	95.71%
**ray1**	29,693,984	28,787,427	98.90%	96.95%	96.49%
**ray2**	29,938,429	28,819,511	98.80%	96.26%	96.20%
**ray3**	27,963,577	26,191,383	98.80%	93.66%	96.28%
**root1**	28,975,018	27,702,311	98.80%	95.61%	90.41%
**root2**	29,955,638	29,045,977	98.80%	96.96%	90.75%
**root3**	28,231,772	26,093,697	98.90%	92.43%	89.78%
**stem1**	29,875,397	28,382,083	98.70%	95%	95.92%
**stem2**	30,062,028	28,718,094	98.80%	95.53%	95.96%
**stem3**	29,076,227	27,702,366	98.80%	95.27%	95.68%

**Fig. 2:**
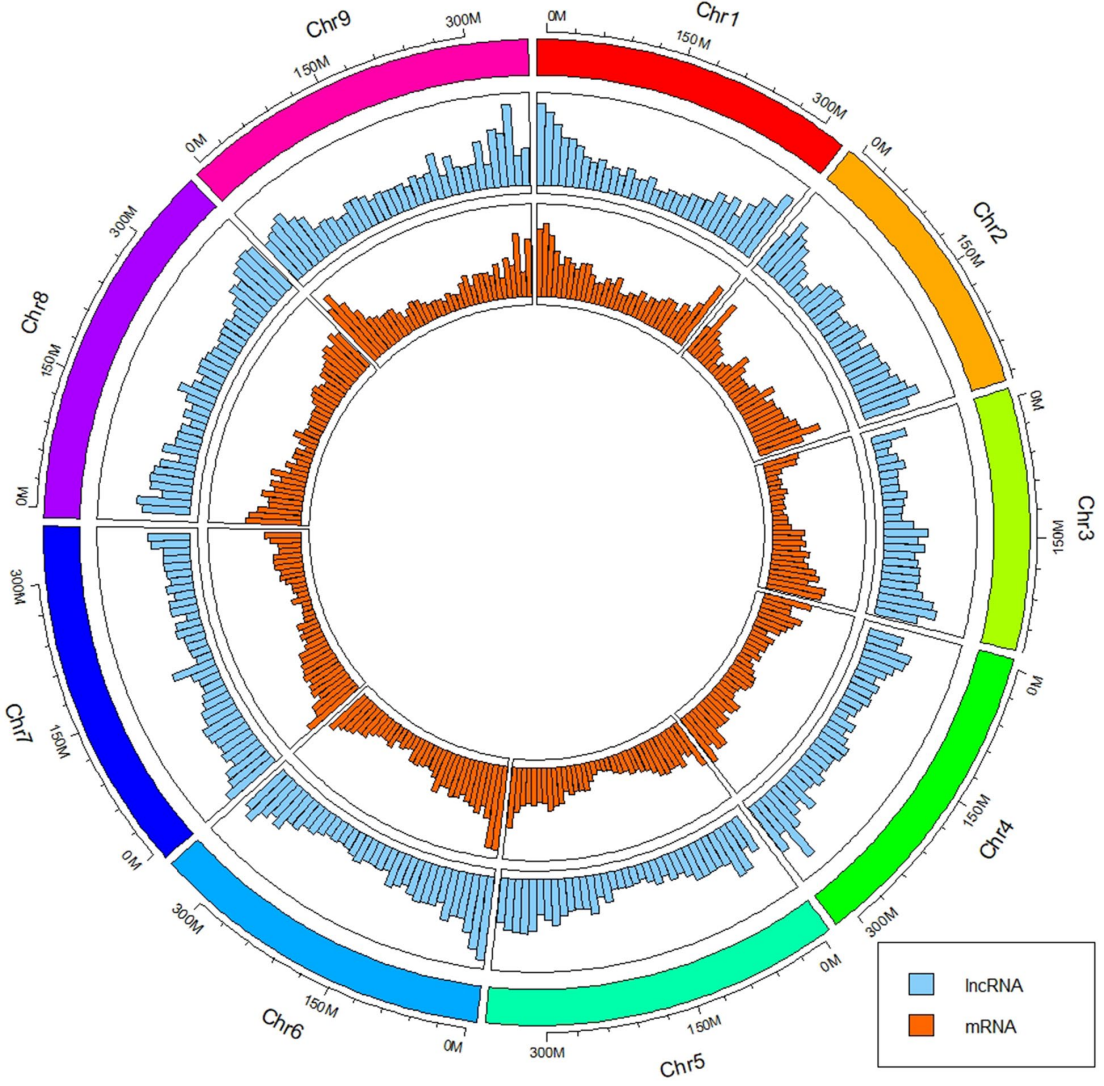
Distribution of mRNAs and lncRNAs on each chromosome in physical bins of 10 Mb (visualized with circlize version 0.4.15 [26])

Following assembly, 501 miRNAs and 22,065 siRNAs were identified in the small RNA library. Except for 74,259 mRNAs identified in *C. seticuspe*, there were 18,265 novel mRNAs and 51,425 novel lncRNAs annotated in the lncRNA and mRNA libraries. The lncRNA and mRNA distribution within chromosomes was analysed, the results of which revealed a more well-distributed location of lncRNAs (Fig. 2). Although the most frequent number of exons for lncRNAs and mRNAs was 1, the average exon number of mRNAs was greater than that of lncRNAs (Fig. S1). The most frequent sequence length distributions of lncRNAs and mRNAs were 0–500 bp and 500–1000 bp, respectively, which indicated that the average length of mRNAs was larger than that of lncRNAs (Fig. S2). The average fragments per kilobase per million mapped reads (FPKM) values of the lncRNAs and mRNAs were 15.7 and 8.9, respectively.

### Expression quantification and comparison of differentially expressed genes (DEGs)

**Fig. 3:**
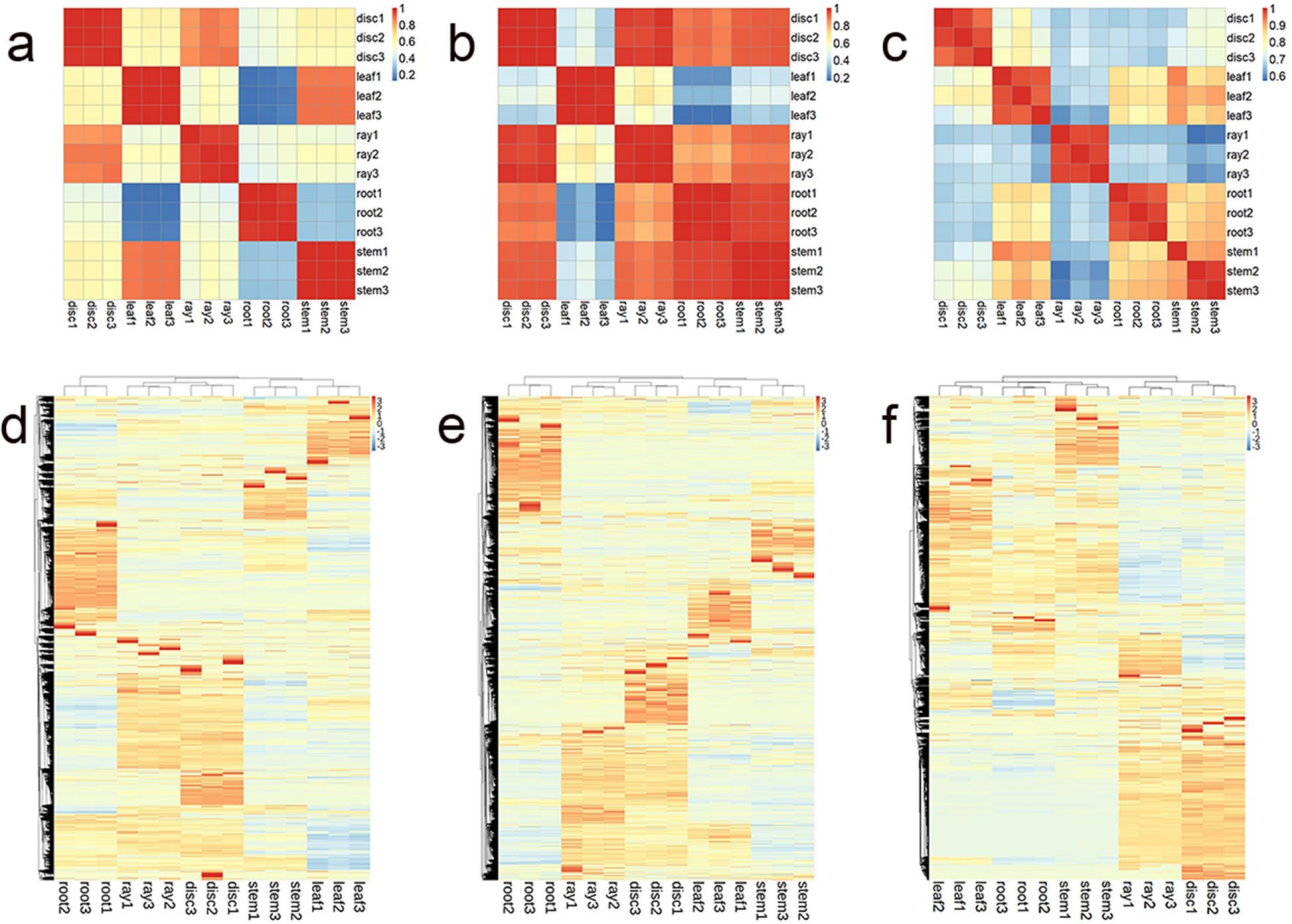
Relevance and expression profiles of mRNAs, lncRNAs and small RNAs among different samples. Calculations and visualizations of Pearson correlation coefficients of mRNAs (**a**), lncRNAs (**b**) and small RNAs (**c**). The expression profiles of mRNAs (**d**), lncRNAs (**e**) and small RNAs (**f**) and the transcripts per million (TPM) and FPKM values were log2 transformed. The heatmap plots were visualized with the R package Pheatmap (version 1.0.12) [27]. Clustering was performed by the hclust method in R

The expression levels of mRNAs, lncRNAs and small RNAs were quantified in different samples. Three biological replicates showed relatively high Pearson correlation coefficients for all three RNAs (Fig. 3 a-c). The clustering analysis of expression profiles also showed a high degree of consistency across biological replicates (Fig. 3 d-f). With respect to mRNAs, the expression profile of disc florets was strongly related to that of ray florets, while the roots were weakly related to the other four organs. Regarding lncRNAs, the expression profile of the leaves was weakly related to the other four organs, which indicated the unique expression patterns of lncRNAs in the leaves. With respect to miRNAs, the expression profile of the leaves was strongly related to that of the roots and stems but was weakly related to that of the ray florets and disc florets. DEGs were identified in different organ comparisons with the following threshold criteria: *p* value ≤0.01 and |log2| ≥ 1. The DEGs of mRNAs and lncRNAs were counted in all comparisons (Fig. S3 and S4). The most significant comparison for both mRNAs and lncRNAs was ‘disc floret vs. root’, in which there were 23,184 and 17,567 DEGs, respectively. The least significant comparison for both mRNAs and lncRNAs was ‘disc floret vs. ray floret’, in which there were 7061 and 5211 DEGs, respectively.

### Expression patterns of flower development-related genes

**Fig. 4:**
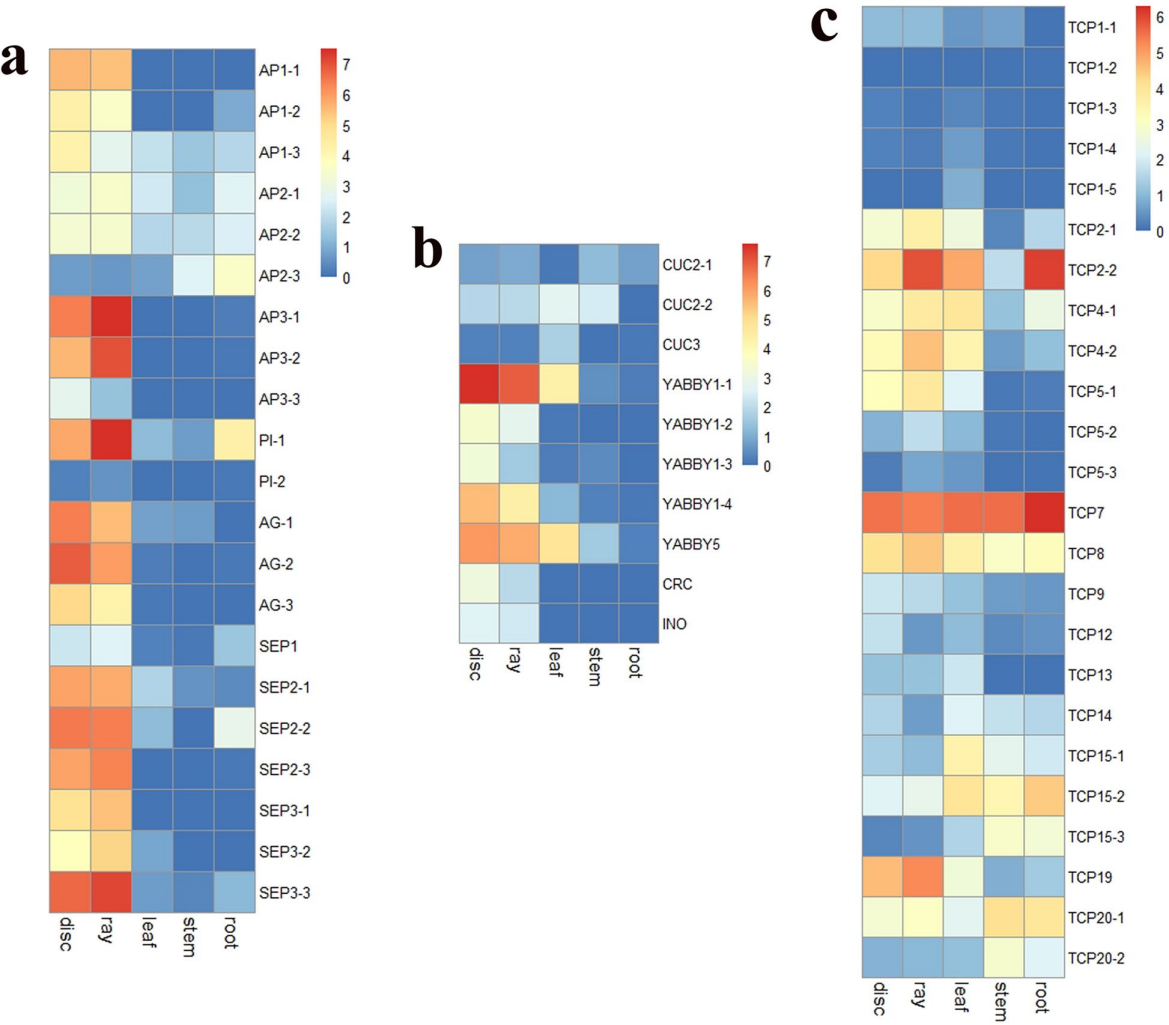
Expression patterns of flower development-related (**a**) *ABCE* genes, (**b**) *CUC* and *YABBY* family genes and (**c**) *TCP* family genes. Bar, log2-transformed FPKM values

To investigate the developmental differences between ray florets and disc florets, we identified flower development-related genes with the BLASTX algorithm by inputting related genes from *Arabidopsis thaliana*. The expression levels of flower development-related genes are shown in Fig. 4. ABCE flower development-related genes displayed higher expression levels in ray florets and disc florets than in the other three organs. Three transcripts (*AP3.1*/CsG_LG4.g53779.1, *AP3.2*/CsG_LG8.g58037.i1 and *PI.1*/CsG_LG9.g33362.i1) of B-class genes had higher transcription in ray florets than in disc florets, similar to the expression of an E-class gene (*SEP3.3*/CsG_LG7.g49236.1) (Fig. 4 a). However, two of the C class genes (*AG.1*/CsG_LG9.g43875.i1 and *AG.2*/CsG_LG7.g04334.i1) showed higher expression levels in the disc florets than in the ray florets. The expression levels of three transcripts of the *CUC* family showed relatively low expression in all five organs. There were higher expression levels of *YABBY1.1*, *YABBY1.4* and *YABBY5* in the disc florets than in the ray florets, suggesting that these genes have a possible regulatory function in the development of flower symmetry (Fig. 4 b). *TCP2.1*, *TCP4.2*, *TCP5.1* and *TCP19* showed relatively high expression levels in the ray florets; however, *TCP2.2* displayed higher expression levels in both the ray florets and the roots (Fig. 4 c). In addition, there was a relatively high expression level of *TCP7* in the roots.

### Network analysis based on the WGCNA results

**Fig. 5:**
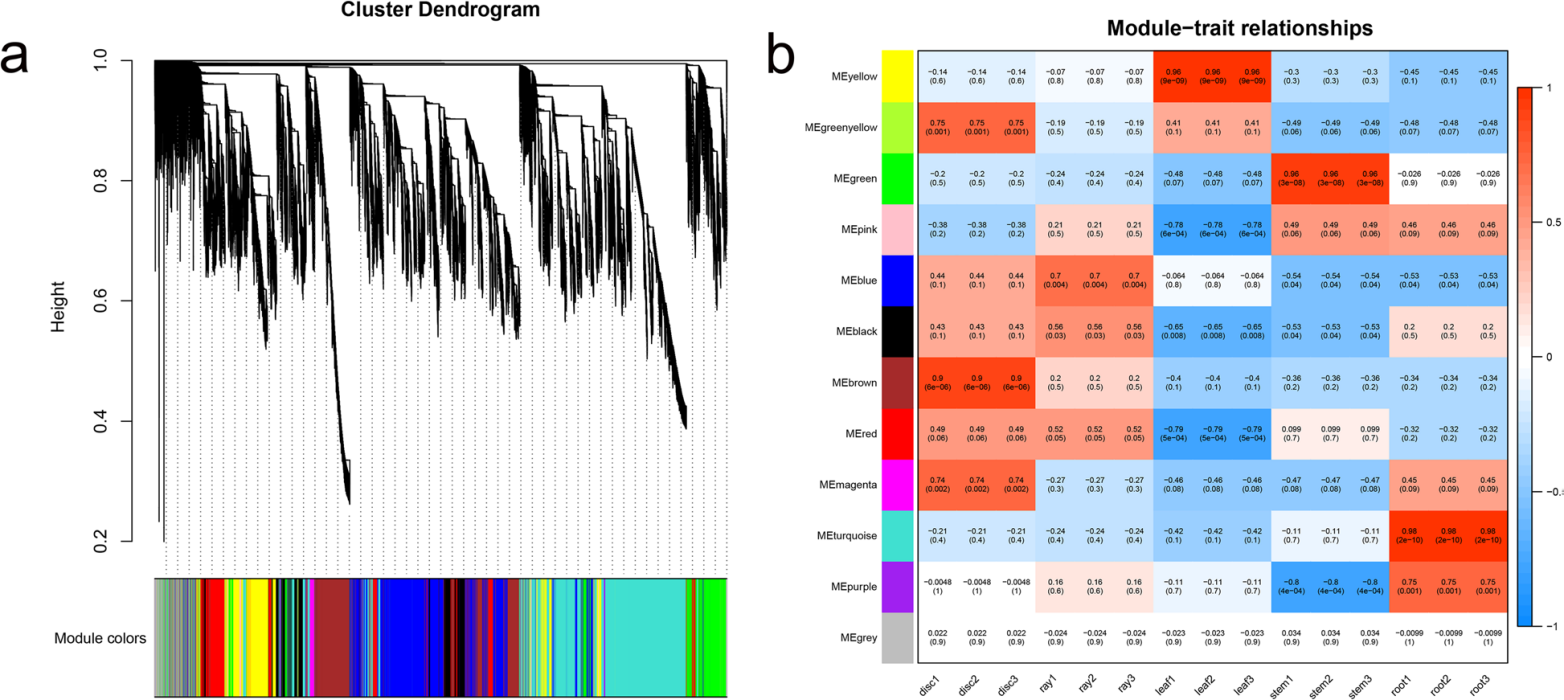
Weighted gene correlation network analysis. **a** Results of a hierarchical cluster analysis performed by WGCNA. Each leaf of the tree represents a single gene, and the tree branches cut to a height of 0.4 produced different modules with specific colours. **b** Relationships between modules and organs. Each column and each row correspond to different organs and modules, respectively. In every box, the upper values are the correlation coefficients, and the lower values are the *p* values

To elucidate the latent regulatory network of *C. seticuspe*, we performed WGCNA to identify the hub genes related to the development of ray florets and disc florets. After clustering analysis was performed, 12 major modules were identified, which were related to different organs (r > 0.7 and *P* < 0.001) (Fig. 5 a and b). In the brown module, there were 8840 genes, which were found to be highly associated with disc florets. The blue module (14,787 genes) was associated with ray florets, the yellow module (7238 genes) was associated with leaves, the green module (6284 genes) was associated with stems, and the turquoise module (20,044 genes) was associated with roots.

The regulatory networks of ray florets and disc florets were constructed with the genes associated with high K_ME_ values in the modules, and the hub mRNAs and lncRNAs are shown in the center of the networks with orange colour; these hubs were selected based on a combination of their FPKM values and gene function (Fig. S5 a and b). In the ray floret-specific module, 3 hub mRNAs and 8 hub lncRNAs were identified. Two of these three genes encoded transcription factors, namely, a MADS-Box family gene (*CMD19*/CsG_LG8.g58037.i1) and a B-BOX gene (*BBX22*/CsG_LG6.g06424.i1). The other gene (*HTH*/CsG_LG5.g56450.i1) encodes a glucose-methanol-choline (GMC) oxidoreductase involved in the biosynthesis of long-chain α-,ω-dicarboxylic fatty acids [28]. The hub lncRNAs were LXLOC_086819, LXLOC_034411, LXLOC_026470, LXLOC_015065, LXLOC_042903, LXLOC_041244, LXLOC_053379 and LXLOC_037271. In the disc floret-specific module, one hub mRNA and three hub lncRNAs were identified. The mRNA was related to a heat shock protein family gene (*HSP70*/MXLOC_096069). The three hub lncRNAs were LXLOC_096252, LXLOC042445 and LXLOC_106636.

### Analysis of ceRNAs and lncNATs of flower development-related genes

**Fig. 6:**
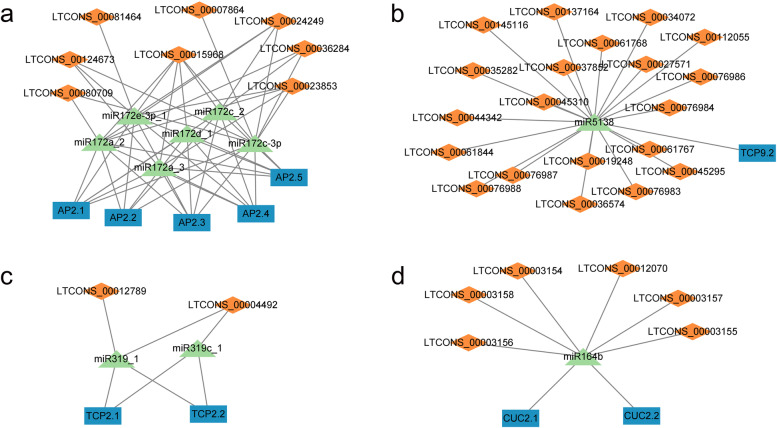
Prediction of ceRNA networks of (**a**) miR172-*AP2*, (**b**) miR5138-*TCP9*, (**c**) miR319-*TCP2* and (**d**) miR164-*CUC2*. The rectangles represent mRNAs, the triangles represent miRNAs, and the diamonds represent lncRNAs

The predicted miRNAs targeting lncRNAs and mRNAs are listed in Table S1. The number of miRNAs targeting lncRNAs was 251, and that of miRNAs targeting mRNAs was 206, of which there were 139 miRNAs targeting both lncRNAs and mRNAs (Fig. S6). To identify the ceRNAs related to flower development, we predicted the miRNAs targeting both flower development-related genes and lncRNAs, the results of which are shown in Fig. 6. Five transcripts of *AP2* (*AP2.1*/CsG_LG3.g24432.i1, *AP2.2*/CsG_LG4.Cse_sc005454.1_g020.1, *AP2.3*/CsG_LG2.g46212.i1, *AP2.4*/MTCONS_00030951 and *AP2.5*/MTCONS_00057150) and 8 lncRNAs were predicted to be targeted by miR172s (Fig. 6 a). In the *TCP* gene family, *TCP9* (*TCP9.2/*MTCONS_00032755) and two transcripts of *TCP2* genes (*TCP2.1*/CsG_LG9.g53441.1 and *TCP2.2*/CsG_LG7.g04758.1) were predicted to be targeted by miR5138 and miR319, respectively (Fig. 6 b and c). Two *CUC2* genes (*CUC2.1*/CsG_LG5.g54126.1 and *CUC2.2*/CsG_LG7.g63595.i1) were predicted to be targeted by miR164b together with 6 lncRNAs (Fig. 6 d).

**Fig. 7:**
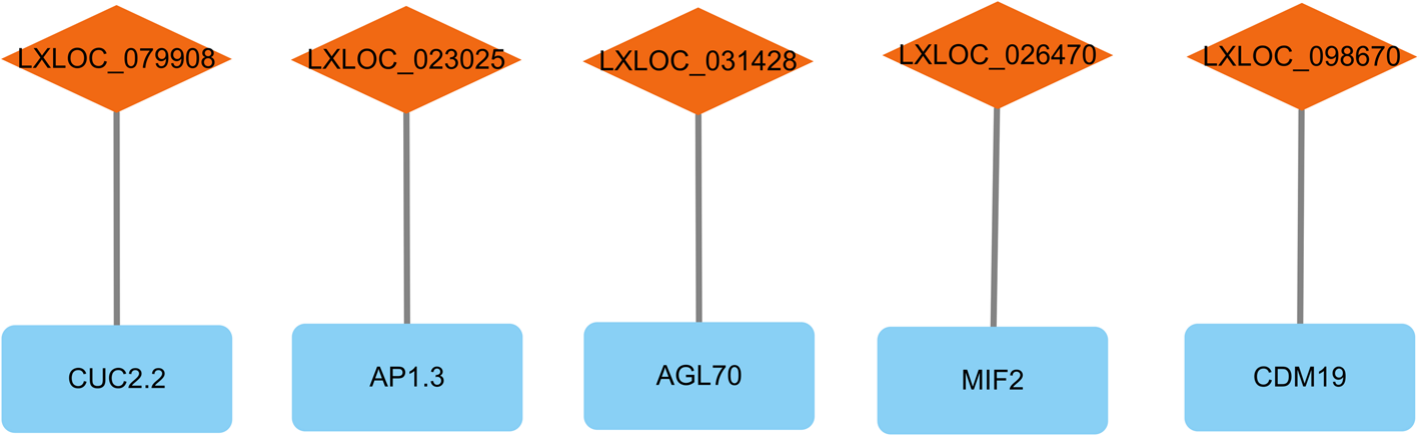
LncNATs of flower development-related genes. The rectangles represent mRNAs, and the diamonds represent lncRNAs

The lncNATs of flower development-related genes were also found to improve the regulatory network (Fig. 7). In the ray floret-specific module, one of the hub lncRNAs (LXLOC_026470) was located in the opposite chain of *MIF2* (CsG_LG3.g28582.1). There was also a lncRNA of the hub mRNA *CDM19* in the ray floret-specific module. There was a lncNAT of *CUC2.2* (CsG_LG7.g63595.i1), which is targeted by miR164 as mentioned above, indicating that there is a complex gene regulatory network involving *CUC2*. There were also two lncNATs (LXLOC_031428 and LXLOC_023025) of *AGL70* (CsG_LG3.g30433) and *AP1.3* (CsG_LG2.g44914.i1).

### qRT–PCR verification of transcriptomic data

**Fig. 8:**
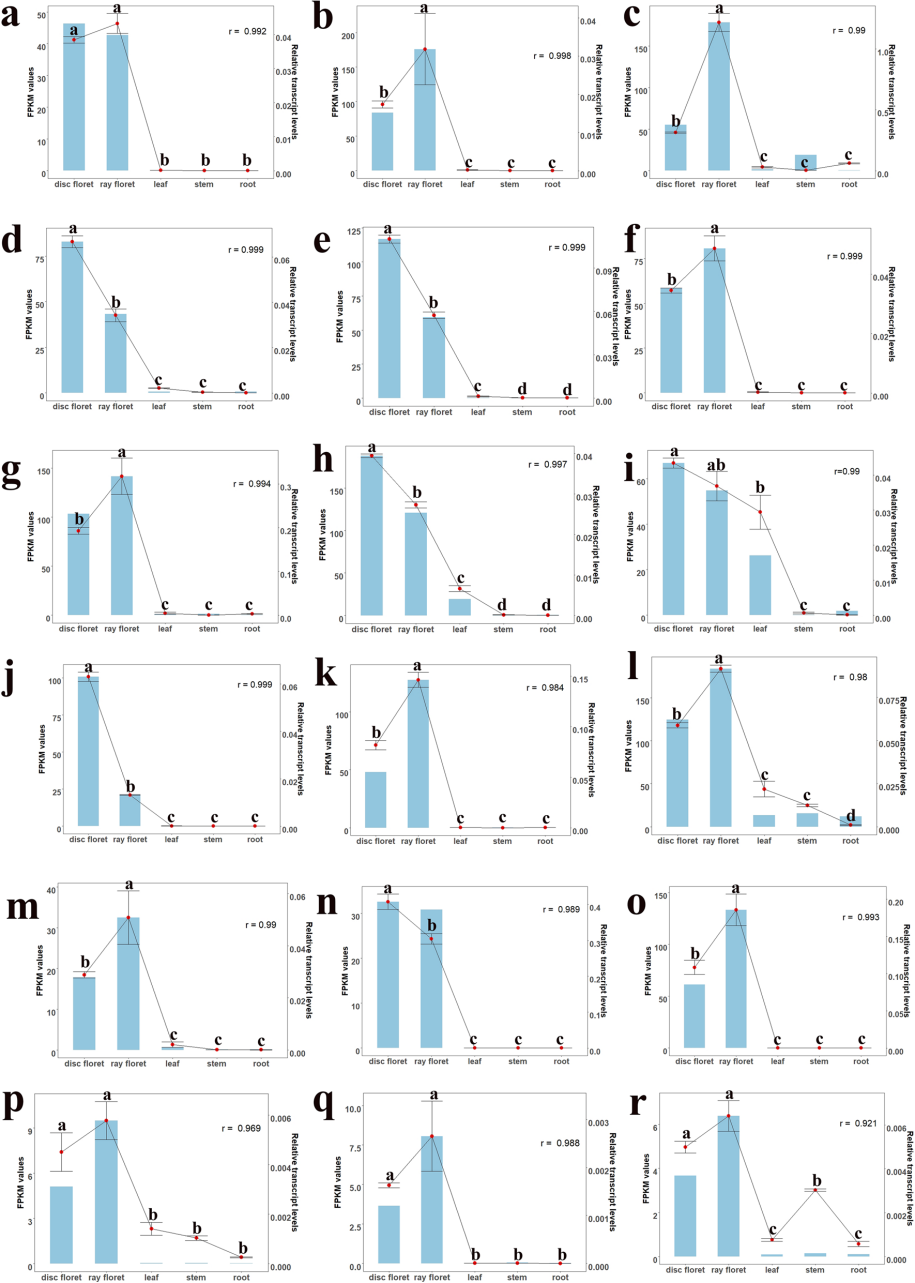
Relative expression levels and FPKM values of flower development-related genes and hub genes in modules; correlation coefficients are shown at the top right. **a** CsG_LG8.g33949.1-*AP1.1*, (**b**) CsG_LG4.g53779.1-*AP3.1*, (**c**) CsG_LG9.g33362.i1-*PI.1*, (**d**) CsG_LG9.g43875.i1-*AG.1*, (**e**) CsG_LG7.g04334.i1-*AG.2*, (**f**) CsG_LG6.g50199.i1-*SEP2.3*, (**g**) CsG_LG7.g49236.1-*SEP3.3*, (**h**) CsG_LG7.g11579.i1-*YABBY1.1*, (**i**) CsG_LG5.g64343.i1-*YABBY5*, (**j**) MTCONS_00042147-*CRC*, (**k**) CsG_LG8.g58037.i1-*CDM19*, (**l**) CsG_LG6.g06424.i1-*BBX22*, (**m**) CsG_LG5.g56450.i1-*HTH*, (**n**) CsG_LG3.g28582.1-*MIF2*, (**o**) LXLOC_026470, (**p**) LXLOC_037271, (**q**) LXLOC_053379, and (**r**) LXLOC_086819. Blue bars represent the mean FPKM values, and red dots represent the mean qRT–PCR values. Error bars indicate the SD for three biological replicates based on qRT–PCR, and shared letters indicate no statistically significant difference between the means (*P* > 0.05) as determined by ANOVA. The correlation coefficient was calculated by R with mean FPKM values and relative transcript levels generated by qRT–PCR

qRT–PCR was performed to verify the FPKM values of flower development-related genes and hub genes in flower-specific modules. As shown in Fig. 8, the FPKM values show a high degree of consistency with the qRT–PCR results, indicating the reliability of the data. Notably, the expression level of *MIF2*, whose lncNAT (LXLOC_026470) is in the ray floret-specific module, was also quantified. The relative expression level of *MIF2* in the disc florets was significantly greater than that in the ray florets, as shown in Fig. 8 n-o. In contrast, the relative expression level of LXLOC_026470 in the ray florets was significantly greater than that in the disc florets. This opposite trend for the relative expression levels suggests that *MIF2* and its lncNAT-LXLOC_026470 might interact to control the development process of ray florets and disc florets.

## Discussion

### Differences in the expression of flower development-related genes between ray florets and disc florets

Petal type is of great importance for both the ornamental and evolutionary value of chrysanthemums. The expression levels of two B-class genes, *AP3* and *PI* homologues (*AP3.1*, *AP3.2* and *PI.1*) of ray florets, were also higher than those of disc florets in both *C. lavandulifolium* and *C. morifolium* [29], the results of which are consistent with the expression patterns in this study (Fig. 4 a). There was a protein–protein interaction between ClAP3 and ClPI in *C*. *lavandulifolium*, illustrating that the formation of heterodimers is needed for proper functioning. In *C. morifolium*, both *AP3* and *PI* have relatively higher expression levels in the petals of ray florets, petals of disc florets and stamens of disc florets [2]. Therefore, B-class genes might play major roles in both petal and stamen development of both types of florets with conserved functions in the *Chrysanthemum* genus. Three of the C-class genes (*AG.1*, *AG.2* and *AG.3*) were more highly transcribed in the disc florets than in the ray florets. The knockdown of two C-class genes (*CAG1* and *CAG2*) in *C. morifolium* resulted in the transformation of reproductive organs into petaloid organs in both disc florets and ray florets, showing that these genes play important roles in the development of pistils and stamens [30]. C-class genes function synergistically with B-class genes to regulate the development of stamens [31]. These findings indicate that B-class and C-class genes might work together to control the development of petals and stamens in *C. seticuspe*. The E-class genes *SEPALLATA* are essential for floral identity; the expression levels of these genes determine the development of ectopic flowers instead of vegetative organs [32]. Most E-class genes were transcribed strongly in floral organs in this study (Fig. 4 a), indicating that these genes play essential roles in flower development. However, one transcript of *SEP3* (*SEP3.3*) had a higher expression level in ray florets than in disc florets. *SEP3* is able to function as a component of various protein complexes [33]. Therefore, the *SEP3.3* protein might interact with other proteins to mediate the development of ray florets and disc florets in *C. seticuspe*.

*YABBY* genes encode proteins that include an N-terminal zinc finger domain and a C-terminal helix-loop-helix motif; these genes are involved in the abaxial domain determination of flower development [34]. In this study, the expression levels of *YABBY* family genes were higher in floral organs than in vegetative organs. Overexpression of *YABBY1* in *C. morifolium* changed petal curvature and inflorescence morphology, of which the petals of ray florets in *CmYAB1*-overexpressing plants tend to be flat [35]. Therefore, the different flower symmetries of disc florets and ray florets in *C. seticuspe* might be related to the differential expression of *YABBY* family genes, which regulate zygomorphic and actinomorphic petal types in ray florets and disc florets, respectively.

### Potential regulatory function of hub genes in ray floret- and disc floret-specific modules

Several hub mRNAs and lncRNAs were identified through WGCNA in both ray floret- and disc floret-specific modules (Fig. S5 a and b). *CDM19* is also an *AP3* homologue and shows a higher expression level in ray florets than in disc florets (Fig. 8 k). The ectopic expression of *CDM19* in *C. morifolium* in Arabidopsis leads to altered carpel development and multi-carpel siliques [36]. Therefore, *CDM19* might control carpel development in *Chrysanthemum*. *BBX22*, a B-box zinc transcription factor, occupied a central position in the ray floret-specific module and had a higher expression level in ray florets than in disc florets (Fig. 8 l). In *C. morifolium*, *BBX20* was identified in the ray petal-specific module*,* and the expression level of *BBX20* was higher in ray florets than in disc florets in six different cultivars [2]. This suggests the conservation of B-box proteins in the regulation of flower morphogenesis in *Chrysanthemum*. *HTH*, encoding a GWC oxidoreductase involved in the biosynthesis of long-chain α-,ω-dicarboxylic fatty acids, participated in floral organ fusion and cuticle membrane structure formation in *Arabidopsis thaliana* [37]. The expression level of *HTH* was higher in ray florets, and *HTH* might control the flower development of ray florets by the biosynthesis pathway of long-chain α-,ω-dicarboxylic fatty acids in *C. seticuspe*. HSP70 is a type of heat shock protein and has been found to participate in cabbage flower development [38], indicating that this protein has a possible role in the regulation of disc floret development. In the ray floret- and disc floret-specific modules, there were not only coding genes but also many lncRNAs, which indicated that they might function together to regulate flower development in *C. seticuspe*.

### ceRNA and lncNAT regulatory networks

LncRNAs can act as ceRNAs and be targeted by miRNAs, which increases the expression levels of mRNAs targeted by miRNAs [39]. In this study, *AP2*, *CUC2* and *TCP* family genes were found to be targeted by miRNAs together with lncRNAs. *AP2* has been implicated as a target of miR172 family members [40], which have been found to regulate the floral transition and flower development in Arabidopsis [41, 42] and the fruit development process in tomato [43]. miR164 can target *CUC2*, the interaction of which influences leaf margin serration in Arabidopsis [44]. In strawberry, the balance between miR164 and *CUC2* was shown to control the serration of leaf margins and flower margins [45]. The balance between miR319 and *TCP* family genes controls the development of the cotyledon boundary and leaf serration, and miR319 and *TCP* genes form complex regulatory networks to determine leaf development when combined with miR164 and *CUC* family genes [46, 47]. In the present study, these miRNAs were predicted to target not only related genes but also lncRNAs. LncRNA-TCONS_00021861 was shown to be targeted by miR528-3p together with *YUCCA7* in rice, and overexpression of TCONS_00021861 led to the sequestration of miR528-3p to upregulate *YUCCA7*, followed by activation of the indole-acetic acid (IAA) biosynthetic pathway [48]. These findings indicate that there is a potential regulatory function of mRNA–miRNA–lncRNA regulatory networks in flower development in *C. seticuspe*.

LncNATs have emerged as pivotal regulators of plant biological processes. In *C. morifolium*, a lncNAT of *TCP1*-lncTCP1 can enhance the expression levels of *TCP1* to improve cold tolerance via the histone modification protein DgATX [49]. The lncNAT of *FLC*-COOLAIR recruits Polycomb repressive complex 2 (PRC2) and deposits H3K27me3 in the chromatin region to facilitate silencing of *FLC* mRNA transcription, which has been shown to affect seed dormancy and flowering time in Arabidopsis [50]. In Chinese cabbage, the lncNAT of *MAPK15* showed involvement in resistance to downy mildew, and there was enhanced expression of *MAPK15* in MSTRG.19915-silenced seedlings [51]. In rice, the lncNAT of *MYB60*-*TWISTED LEAF* might recruit H3K27me3 to silence the expression of MYB60, controlling twisted leaf blades [20]. In this study, we identified several flower development-related genes and their lncNATs, of which the expression levels of *MIF2* and its lncNAT-LXLOC_026470 tended to be the opposite in ray florets and disc florets. The positional relationship of *MIF2* and LXLOC_026470 on chromosome 3 is shown in Fig. S7. *The MIF* gene family encodes small zinc finger proteins, and overexpression of two homologues of *MIF2* (*MIF1* and *MIF3*) has been shown to influence the determinate leaf growth of Arabidopsis [52]. Together with TOPLESS and HISTONE DEACETYLASE 19, the MIF2 homologue in tomato-SlIMA recruits SlKNU to form a transcriptional repressor complex, which represses the expression of *SlWUS*, participates in the floral meristem termination process and ultimately determines carpel number [53]. Therefore, LXLOC_026470 may recruit histone proteins to silence the expression of *MIF2* to influence the development of ray florets and disc florets in *C. seticuspe*.

## Conclusion

Through whole-transcriptome sequencing of *C. seticuspe*, annotation of the genome has been improved with information concerning mRNAs, lncRNAs and small RNAs. mRNA–miRNA–lncRNA networks were constructed to increase our understanding of ray floret and disc floret development in chrysanthemums. We identified a lncNAT-mRNA combination, LXLOC_026470 and *MIF2*, that might be related to flower development of the capitulum via histone modification.

## Methods

### Plant materials

Seedlings of Gojo-0 were obtained from the Graduate School of Integrated Sciences for Life, Hiroshima University, 1–4-3, Kagamiyama, Higashi-Hiroshima, Japan [6]. The seedlings were planted in the greenhouse of Nanjing Agricultural University, and the growing environment was the same as that described by Nakano et al. [54]. The leaves, stems and roots were sampled from plants at the vegetative stage, while the ray florets and disc florets were sampled from plants at the flowering stage.

### Small RNA library construction, sequencing and data processing

Total RNA was extracted with TRIzol reagent (Invitrogen, Carlsbad, CA, USA) according to the provided protocol. Extracted RNA was used to construct a small RNA library for each sample. After electrophoretic separation on a 15% urea denaturing polyacrylamide gel electrophoresis (PAGE) gel, the bands of the 18–30 nt small RNA region were recovered. After being adenylated with 3′ adapters annealed to unique molecular identifiers (UMIs) and ligated with 5′ adapters, the small RNAs were transcribed into cDNA via SuperScript II Reverse Transcriptase (Invitrogen, USA). cDNA fragments were enriched using several rounds of PCR amplification. The PCR products were selected by agarose gel electrophoresis with target fragments of 110 ~ 130 bp and then purified by a QIAquick Gel Extraction Kit (QIAGEN, Valencia, CA). The small RNA libraries were subsequently sequenced on the MGISEQ-2000RS platform (BGI-Shenzhen, China).

The raw data of small RNA libraries were considered raw tags. The raw tags were filtered by removing unqualified tags (tags of law quality, tags with 5′ primer contaminants, tags without 3′ primers, tags without insertions, tags with poly(A) nucleotides and tags shorter than 18 nt). After filtering, the clean tags were mapped to the reference genome sequence and sequences in other sRNA databases, including miRbase [27], siRNA sequences [28] and snoRNA sequences [29], with Bowtie2 [30]. The quantification of small RNA expression was performed by counting the absolute numbers of molecules with unique molecular identifiers [31].

### mRNA and lncRNA library construction, sequencing and data processing

The RNA used in the lncRNA and mRNA libraries was the same as the RNA used in the small RNA libraries. However, 1 μg of total RNA was purified with a Ribo-Zero™ Magnetic Kit (Plant Leaf, Epicentre) to deplete the rRNA. The purified RNA was fragmented by adding First Strand Master Mix (Invitrogen, USA). Subsequently, RNA was transcribed into first-strand cDNA using random primers, followed by second-strand cDNA synthesis. The synthesized cDNA was then subjected to end-repair and 3′ adenylation. The ends of these 3′-adenylated cDNA fragments were subsequently ligated with adapters. The cDNA fragments were enriched using several rounds of PCR amplification with PCR Primer Cocktail and PCR Mix. Then, the PCR products were purified with AMPure XP Beads. The resulting mRNA and lncRNA libraries were sequenced on the BGISEQ-500 platform (BGI-Shenzhen, China).

The raw data of the lncRNA and mRNA libraries were assessed with SOAPnuke [32] by (1) removing reads containing sequencing adapters; (2) removing reads whose low-quality base percentage (base quality less than or equal to 5) was more than 20%; and (3) removing reads whose unknown base (‘N’ base) percentage was more than 5%. Subsequently, the clean reads of the lncRNA and mRNA libraries were aligned using HISAT [33] and assembled using StringTie [34] and Cufflinks [35]. The coding availability was assessed by CPC [36], txCdsPredict (https://github.com/ENCODE-DCC/kentUtils/tree/master/src/hg/txCds/txCdsPredict), CNCI [37] and the Pfam database [38]. Only when at least three of the four judgement methods were consistent could we confirm that a transcript was an mRNA or lncRNA. The quantification of transcripts was transformed into FPKM values for lncRNA and mRNA libraries using RSEM [39]. A heatmap was constructed by Pheatmap according to the gene expression patterns in different samples.

### Prediction of miRNA targeting

The prediction results are shown in Table S1. Software including psRobot [55] and TargetFinder [56] was used to predict the target genes of the miRNAs. Functional annotations of targeted mRNAs were performed with the NCBI-NR database and Gene Ontology (GO) [57].

### WGCNA and network visualization

The transcriptome profiles of biological replicates were also calculated to obtain a comprehensive regulatory network. The FPKM values were log2 normalized and then input into the WGCNA package version 1.70–3 [58] in R. The outliers were removed to standardize the whole dataset. A soft threshold power of 18 was used to construct the adjacency matrix. The dynamic tree was cut with a minimum module size of 30 and a merging threshold of 0.25. Each organ was defined as a trait, and the correlations between modules and traits were analysed using Pearson correlation coefficients (PCCs) [59]. The genes with a high K_ME_ were identified as hub genes [60]. The whole-gene coexpression, ceRNA and lncRNA network was visualized by Cytoscape v3.9.1 software (https://cytoscape.org/).

### qRT–PCR

RNA was extracted from the five organs as mentioned above. The primer pairs designed and used for qRT–PCR are listed in Table S2. cDNA was synthesized using a PrimeScript RT Reagent Kit (TaKaRa, Japan), and qRT–PCR was performed with a SYBR Premix Ex Taq II Kit (TaKaRa, Japan) according to the manufacturer’s protocol on a 480 Real-Time PCR System (Roche, Switzerland). Three biological replicates were included, of which three technical replicates were used for each biological replicate. The reference gene *CsEF1α* was used to normalize the expression levels using the 2^−ΔΔCT^ method [61].

## Supplementary Information

**Additional file 1.**

**Additional file 2.**

**Additional file 3.**

## Data Availability

The relevant data in this study are included in this article and the supplementary files. The transcriptome data have been uploaded to the National Center for Biotechnology Information under accession number PRJNA82048835 (https://dataview.ncbi.nlm.nih.gov/object/PRJNA820488?reviewer=t9sj9hjp5oetuede4ka11lsjfd).

## References

[1] Jeffrey C (2007). Compositae: introduction with key to tribes. Families and Genera of Vascular Plants.

[2] Ding L, Song A, Zhang X, Li S, Su J, Xia W, at el. (2020). The core regulatory networks and hub genes regulating flower development in *Chrysanthemum morifolium*. Plant Mol Biol.

[3] Song A, Gao T, Li P, Chen S, Guan Z, Wu D, at el. (2016). Transcriptome-wide identification and expression profiling of the DOF transcription factor gene family in *Chrysanthemum morifolium*. Front Plant Sci.

[4] Song C, Liu Y, Song A, Dong G, Zhao H, Sun W, at el. (2018). The *Chrysanthemum nankingense* genome provides insights into the evolution and diversification of chrysanthemum flowers and medicinal traits. Mol Plant.

[5] Hirakawa H, Sumitomo K, Hisamatsu T, Nagano S, Shirasawa K, Higuchi Y, at el. (2019). *De novo* whole-genome assembly in *Chrysanthemum seticuspe*, a model species of chrysanthemums, and its application to genetic and gene discovery analysis. DNA Res.

[6] Nakano M, Hirakawa H, Fukai E, Toyoda A, Kajitani R, Minakuchi Y, at el. (2021). A chromosome-level genome sequence of *Chrysanthemum seticuspe*, a model species for hexaploid cultivated chrysanthemum. Commun Biol.

[7] Krizek BA, Fletcher JC (2005). Molecular mechanisms of flower development: an armchair guide. Nat Rev Genet.

[8] Aida R, Komano M, Saito M, Nakase K, Murai K (2008). Chrysanthemum flower shape modification by suppression of chrysanthemum*-AGAMOUS* gene. Plant Biotechnol.

[9] Hileman LC (2014). Bilateral flower symmetry—how, when and why?. Curr Opin Plant Biol.

[10] Juntheikki-Palovaara I, Tahtiharju S, Lan TY, Broholm SK, Rijpkema AS, Ruonala R, at el. (2014). Functional diversification of duplicated CYC2 clade genes in regulation of inflorescence development in *Gerbera hybrida* (Asteraceae). Plant J.

[11] Shen CZ, Chen J, Zhang CJ, Rao GY, Guo YP (2021). Dysfunction of *CYC2g* is responsible for the evolutionary shift from radiate to disciform flowerheads in the *Chrysanthemum* group (Asteraceae: anthemideae). Plant J.

[12] Xu X, Smaczniak C, Muino JM, Kaufmann KJ (2021). Cell identity specification in plants: lessons from flower development. J Exp Bot.

[13] Bowman JL (2000). The YABBY gene family and abaxial cell fate. Curr Opin Plant Biol.

[14] Sampath K, Ephrussi A (2016). CncRNAs: RNAs with both coding and non-coding roles in development. Development..

[15] Yu Y, Zhang Y, Chen X, Chen Y (2019). Plant noncoding RNAs: hidden players in development and stress responses. Annu Rev Cell Dev Biol.

[16] Willmann MR, Poethig RS (2007). Conservation and evolution of miRNA regulatory programs in plant development. Curr Opin Plant Biol.

[17] Damodharan S, Corem S, Gupta SK, Arazi TJ (2018). Tuning of *SlARF10A* dosage by sly-miR160a is critical for auxin-mediated compound leaf and flower development. Plant J.

[18] Kim ED, Sung S (2012). Long noncoding RNA: unveiling hidden layer of gene regulatory networks. Trends Plant Sci.

[19] Ariel F, Romero-Barrios N, Jégu T, Benhamed M, Crespi M (2015). Battles and hijacks: noncoding transcription in plants. Trends Plant Sci.

[20] Liu X, Li D, Zhang D, Yin D, Zhao Y, Ji C, at el. (2018). A novel antisense long noncoding RNA, T*WISTED LEAF*, maintains LEAF blade flattening by regulating its associated sense R2R3-MYB gene in rice. New Phytol.

[21] Zhou Y-F, Zhang Y-C, Sun Y-M, Yu Y, Lei M-Q, Yang Y-W, at el. (2021). The parent-of-origin lncRNA *MISSEN* regulates rice endosperm development. Nat Commun.

[22] Jiang N, Cui J, Shi Y, Yang G, Zhou X, Hou X, et al. Tomato lncRNA23468 functions as a competing endogenous RNA to modulate *NBS-LRR* genes by decoying miR482b in the tomato-*Phytophthora infestans* interaction. Hortic Res. 2019:6. 10.1038/s41438-018-0096-0 PMC6355781 30729018

[23] Liu S, Wu L, Qi H, Xu M (2019). LncRNA/circRNA–miRNA–mRNA networks regulate the development of root and shoot meristems of *Populus*. Ind Crop Prod.

[24] Tu Z, Xia H, Yang L, Zhai X, Shen Y, Li H. The roles of microRNA-long non-coding RNA-mRNA networks in the regulation of leaf and flower development in *Liriodendron chinense*. Front Plant Sci. 2022:13. 10.3389/fpls.2022.816875 PMC8829146 35154228

[25] Wen X, Qi S, Yang L, Hong Y, Dai S (2019). Expression pattern of candidate genes in early capitulum morphogenesis of *Chrysanthemum lavandulifolium*. Sci Hortic.

[26] Gu Z, Gu L, Eils R, Schlesner M, Brors B (2014). *Circlize* implements and enhances circular visualization in R. Bioinformatics..

[27] Kolde R. pheatmap: Pretty Heatmaps. R package version 1.0.12. 2019, 8.

[28] Xu Y, Liu S, Liu Y, Ling S, Chen C, Yao J (2017). HOTHEAD-like *HTH1* is involved in anther cutin biosynthesis and is required for pollen fertility in rice. Plant Cell Physiol.

[29] Wen X, Qi S, Huang H, Wu X, Zhang B, Fan G, at el. (2019). The expression and interactions of ABCE-class and *CYC2-like* genes in the capitulum development of *Chrysanthemum lavandulifolium* and *C.× morifolium*. Plant Growth Regul.

[30] Sasaki K, Yoshioka S, Aida R, Ohtsubo N (2021). Production of petaloid phenotype in the reproductive organs of compound flowerheads by the co-suppression of class-C genes in hexaploid *Chrysanthemum morifolium*. Planta..

[31] Wang Q, Dan N, Zhang X, Lin S, Bao M, Fu X. Identification, characterization and functional analysis of C-class genes associated with double flower trait in carnation (*Dianthus caryphyllus* L.). Plants. 2020;9(1):87. 10.3390/plants9010087 PMC7020439 31936710

[32] Ditta G, Pinyopich A, Robles P, Pelaz S, Yanofsky MF (2004). The *SEP4* gene of *Arabidopsis thaliana* functions in floral organ and meristem identity. Curr Biol.

[33] Immink RG, Tonaco IA, de Folter S, Shchennikova A, van Dijk AD, Busscher-Lange J, at el. (2009). SEPALLATA3: the 'glue' for MADS box transcription factor complex formation. Genome Biol.

[34] Finet C, Floyd SK, Conway SJ, Zhong B, Scutt CP, Bowman JL (2016). Evolution of the YABBY gene family in seed plants. Evol Dev.

[35] Ding L, Zhao K, Zhang X, Song A, Su J, Hu Y, at el. (2019). Comprehensive characterization of a floral mutant reveals the mechanism of hooked petal morphogenesis in *Chrysanthemum morifolium*. Plant Biotechnol J.

[36] Githeng’u SK, Ding L, Zhao K, Zhao W, Chen S, Jiang J, at el. (2020). Ectopic expression of Chrysanthemum *CDM19* in *Arabidopsis* reveals a novel function in carpel development. Electron J Biotechnol.

[37] Kurdyukov S, Faust A, Trenkamp S, Bär S, Franke R, Efremova N, at el. (2006). Genetic and biochemical evidence for involvement of *HOTHEAD* in the biosynthesis of long-chain α-, ω-dicarboxylic fatty acids and formation of extracellular matrix. Planta..

[38] Su H, Xing M, Liu X, Fang Z, Yang L, Zhuang M, at el. (2019). Genome-wide analysis of *HSP70* family genes in cabbage (*Brassica oleracea* var. *capitata*) reveals their involvement in floral development. BMC Genomics.

[39] Tay Y, Rinn J, Pandolfi PP (2014). The multilayered complexity of ceRNA crosstalk and competition. Nature..

[40] Aukerman MJ, Sakai H (2003). Regulation of flowering time and floral organ identity by a microRNA and its *APETALA2*-like target genes. Plant Cell.

[41] Ó’Maoiléidigh DS, van Driel AD, Singh A, Sang Q, Le Bec N, Vincent C, at el. (2021). Systematic analyses of the *MIR172* family members of *Arabidopsis* define their distinct roles in regulation of *APETALA2* during floral transition. PLoS Biol.

[42] Wollmann H, Mica E, Todesco M, Long JA, Weigel D (2010). On reconciling the interactions between *APETALA2*, miR172 and *AGAMOUS* with the ABC model of flower development. Development..

[43] Chung M-Y, Nath UK, Vrebalov J, Gapper N, Lee JM, Lee D-J, at el. (2020). Ectopic expression of miRNA172 in tomato (*Solanum lycopersicum*) reveals novel function in fruit development through regulation of an *AP2* transcription factor. BMC Plant Biol.

[44] Nikovics K, Blein T, Peaucelle A, Ishida T, Morin H, Aida M, at el. (2006). The balance between the *MIR164A* and *CUC2* genes controls leaf margin serration in *Arabidopsis*. Plant Cell.

[45] Zheng G, Wei W, Li Y, Kan L, Wang F, Zhang X, at el. (2019). Conserved and novel roles of *miR164-CUC2* regulatory module in specifying leaf and floral organ morphology in strawberry. New Phytol.

[46] Koyama T, Sato F, Ohme-Takagi M (2017). Roles of miR319 and TCP transcription factors in leaf development. Plant Physiol.

[47] Bresso EG, Chorostecki U, Rodriguez RE, Palatnik JF, Schommer C (2018). Spatial control of gene expression by miR319-regulated TCP transcription factors in leaf development. Plant Physiol.

[48] Chen J, Zhong Y, Qi X (2021). LncRNA TCONS_00021861 is functionally associated with drought tolerance in rice (*Oryza sativa* L.) via competing endogenous RNA regulation. BMC Plant Biol.

[49] Li X, Yang Q, Liao X, Tian Y, Zhang F, Zhang L, et al. A natural antisense RNA improves chrysanthemum cold tolerance by regulating the transcription factor *DgTCP1*. Plant Physiol. 2022. 10.1093/plphys/kiac267 PMC9434197 35728057

[50] Chen M, Penfield S (2018). Feedback regulation of COOLAIR expression controls seed dormancy and flowering time. Science..

[51] Zhang B, Su T, Li P, Xin X, Cao Y, Wang W, et al. Identification of long noncoding RNAs involved in resistance to downy mildew in Chinese cabbage. Hortic. Res. 2021:8. 10.1038/s41438-021-00479-1 PMC7917106 33642586

[52] Hu W, Feng B, Ma H (2011). Ectopic expression of the Arabidopsis *MINI ZINC FINGER1* and *MIF3* genes induces shoot meristems on leaf margins. Plant Mol Biol.

[53] Bollier N, Sicard A, Leblond J, Latrasse D, Gonzalez N, Gévaudant F, at el. (2018). At-MINI ZINC FINGER2 and Sl-INHIBITOR OF MERISTEM ACTIVITY, a conserved missing link in the regulation of floral meristem termination in Arabidopsis and tomato. Plant Cell.

[54] Nakano M, Taniguchi K, Masuda Y, Kozuka T, Aruga Y, Han J, at el. (2019). A pure line derived from a self-compatible *Chrysanthemum seticuspe* mutant as a model strain in the genus *Chrysanthemum*. Plant Sci.

[55] Wu H-J, Ma Y-K, Chen T, Wang M, Wang X-J (2012). PsRobot: a web-based plant small RNA meta-analysis toolbox. Nucleic Acids Res.

[56] Fahlgren N, Carrington JC. miRNA target prediction in plants. Plant MicroRNAs. 2010:51–7. 10.1007/978-1-60327-005-2_4 19802588

[57] Gene Ontology Consortium (2004). The gene ontology (GO) database and informatics resource. Nucleic Acids Res.

[58] Langfelder P, Horvath S (2008). WGCNA: an R package for weighted correlation network analysis. BMC Bioinformatics.

[59] Du J, Wang S, He C, Zhou B, Ruan Y-L, Shou HJ (2017). Identification of regulatory networks and hub genes controlling soybean seed set and size using RNA sequencing analysis. J Exp Bot.

[60] Langfelder P, Mischel PS, Horvath S (2013). When is hub gene selection better than standard meta-analysis?. PLoS One.

[61] Livak KJ, Schmittgen TD (2001). Analysis of relative gene expression data using real-time quantitative PCR and the 2^−ΔΔCT^ method. Methods..

