# Supplementary material for: Whole-transcriptome profiles of Chrysanthemum seticuspe improve genome annotation and shed new light on mRNA–miRNA–lncRNA networks in ray florets and disc florets
Source: BMC Plant Biol. 2022 Nov 5;22:515. doi: 10.1186/s12870-022-03889-y (PMC9636758; doi:10.1186/s12870-022-03889-y)
Supplement: Supplementary file 1 — Additional file 1. [file 12870_2022_3889_MOESM1_ESM.docx]

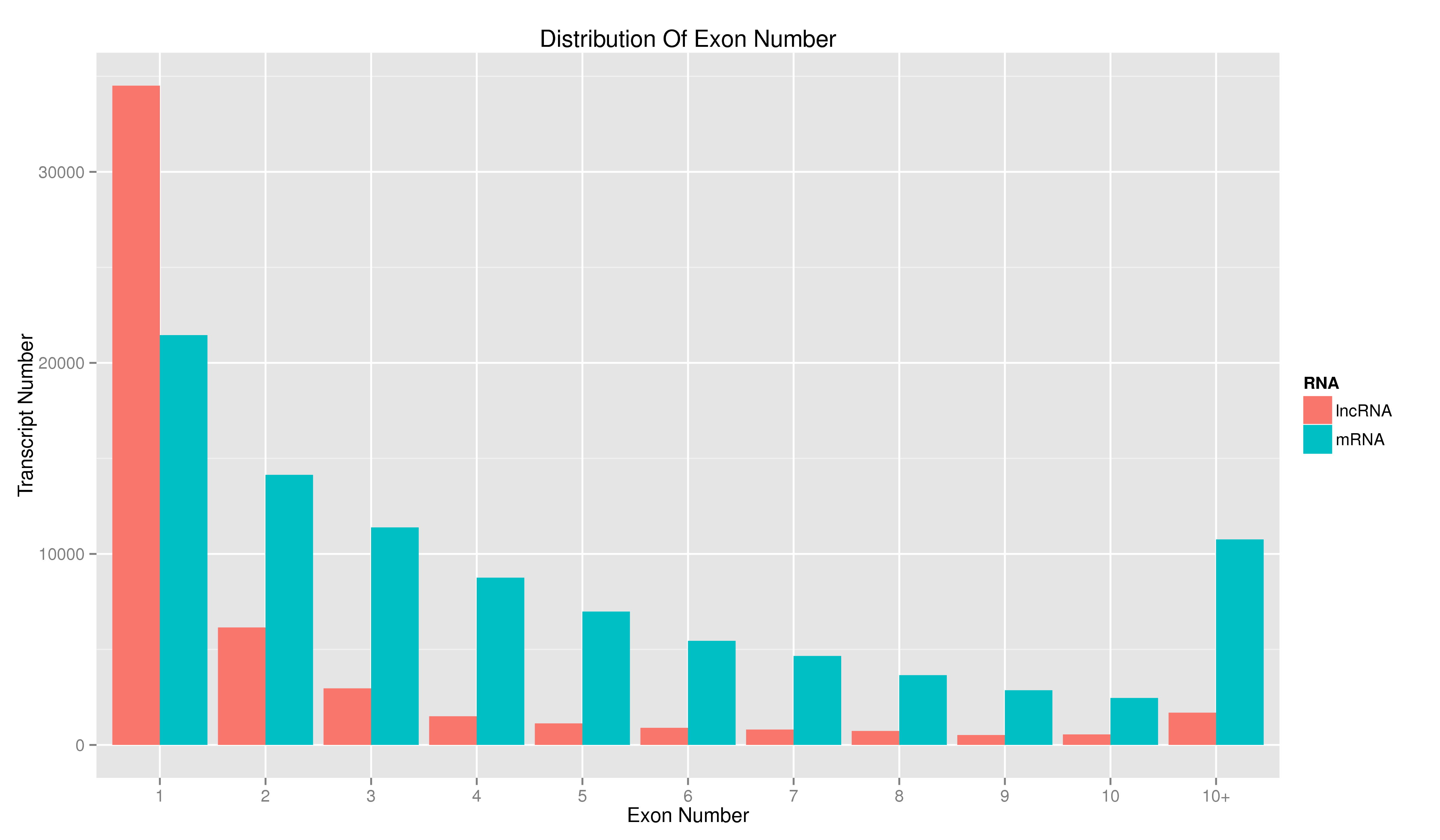


**Fig. S1** The distribution of exon number of lncRNA and mRNA.


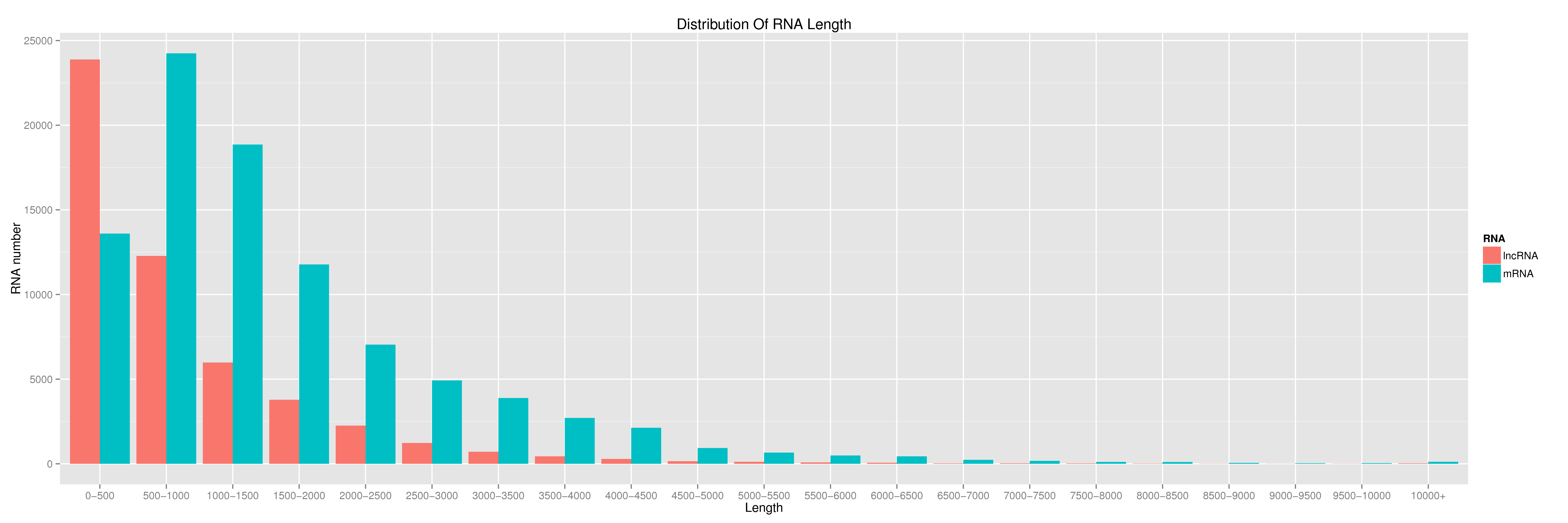


**Fig. S2** The distribution of transcript length of lncRNA and mRNA.


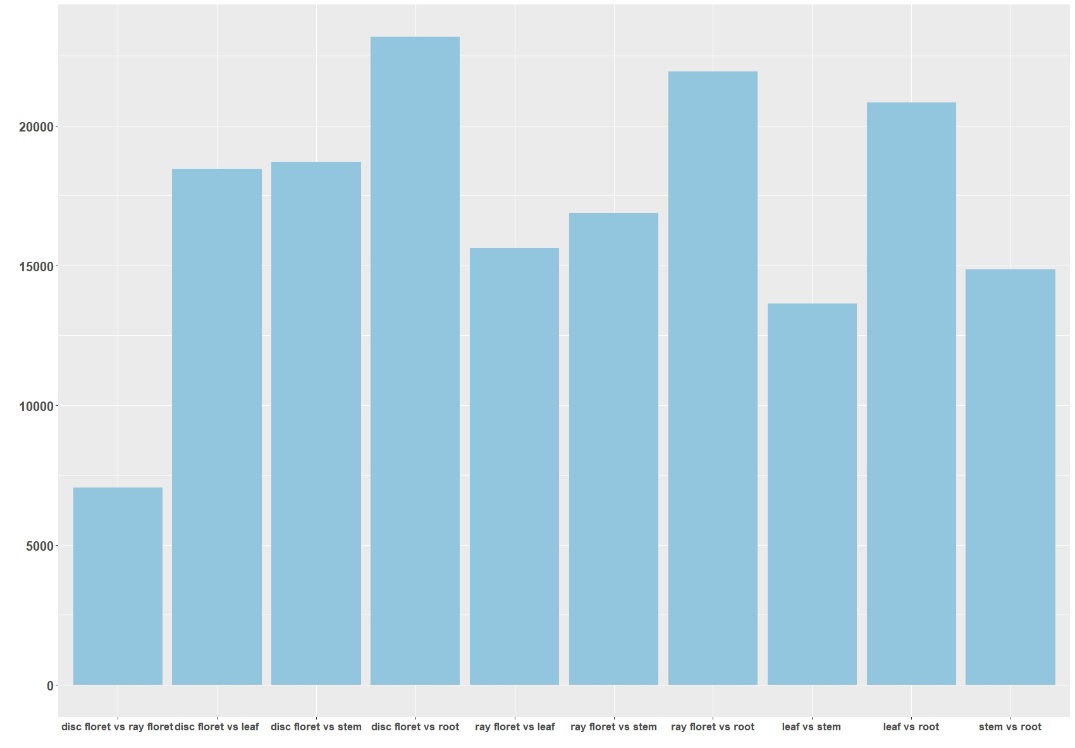


**Fig. S3** The number of DEGs of mRNA among ten comparation groups of five organs.


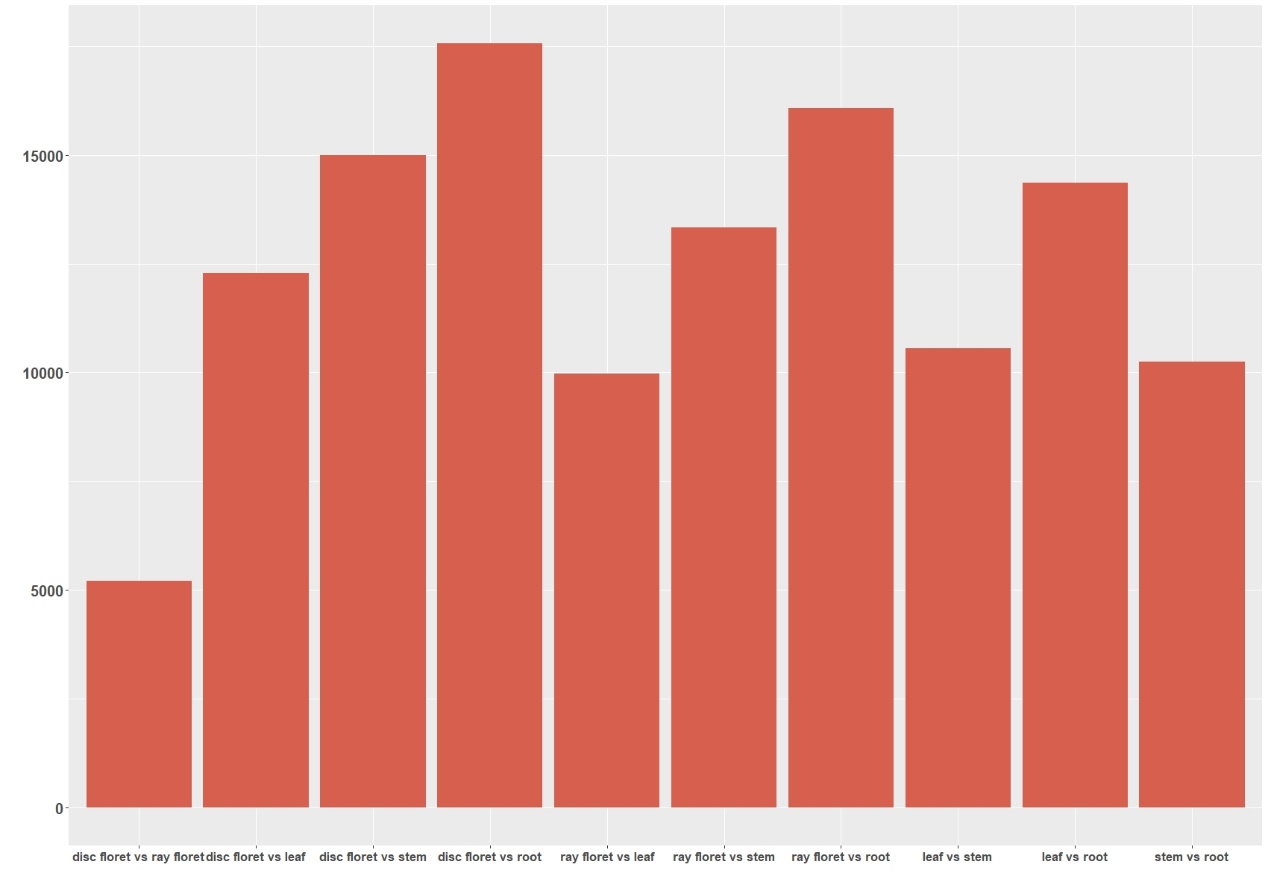


**Fig. S4** The number of DEGs of lncRNA among ten comparation groups of five organs.


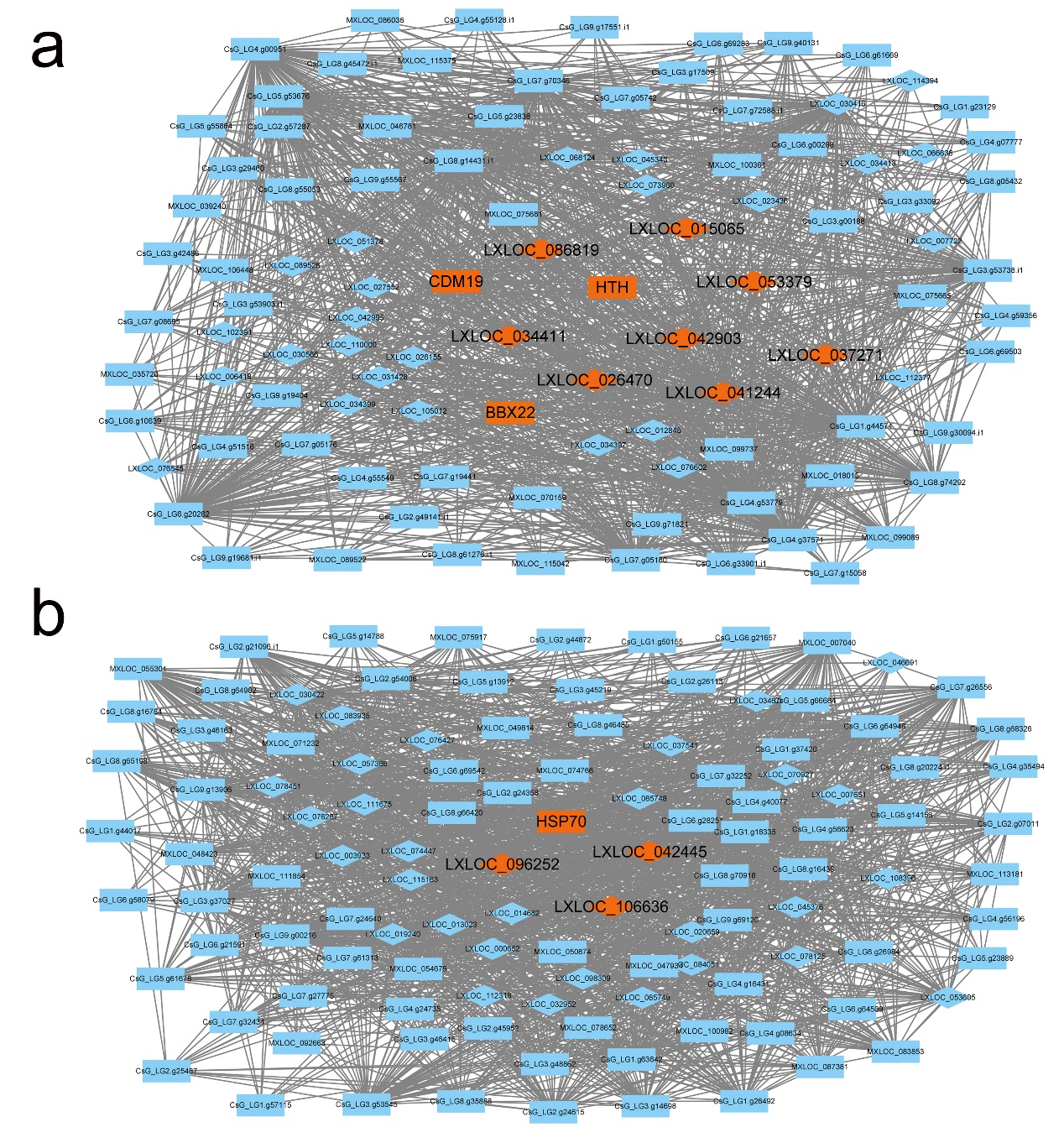


**Fig. S5** Correlation network visualization of ray florets (a) and disc florets (b). The rectangles represent mRNAs, and the diamonds represent lncRNAs. The hub genes are colored orange, with other genes shown in blue.


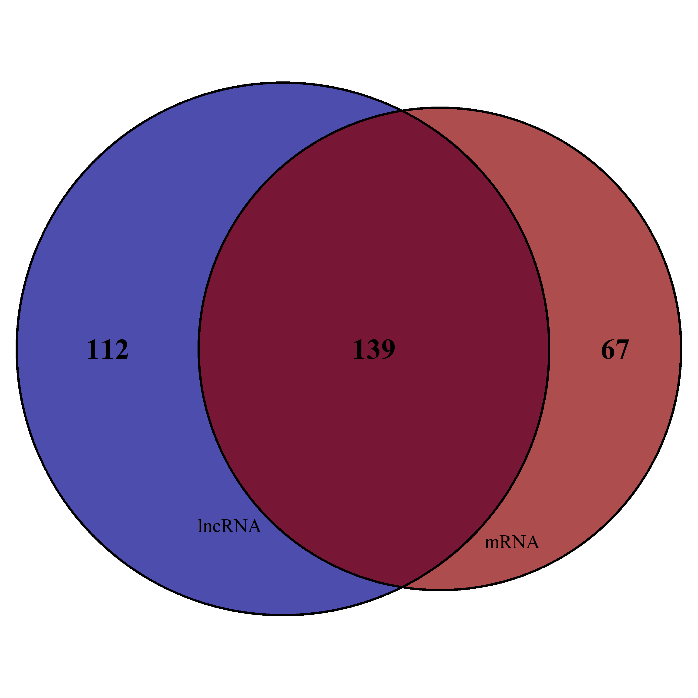


**Fig. S6** A Venn diagram of the number of miRNAs targeting lncRNAs and mRNAs.


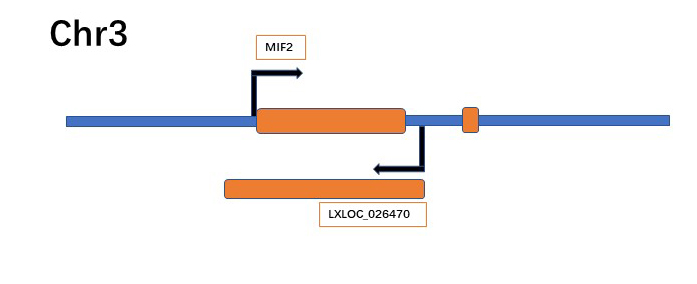


**Fig. S7** The location of *MIF2* and its lncNAT-LXLOC_026470 on chromosome 3. *MIF2* is located in the minus strain, and LXLOC_026470 is located in plus strain from the in the intron of *MIF2*. Orange bars represent exons, blue bars represent chromosome 3 sequence and black arrows represent the direction of transcription.
