# Supplementary material for: Whole-transcriptome profiles of Chrysanthemum seticuspe improve genome annotation and shed new light on mRNA–miRNA–lncRNA networks in ray florets and disc florets
Source: BMC Plant Biol. 2022 Nov 5;22:515. doi: 10.1186/s12870-022-03889-y (PMC9636758; doi:10.1186/s12870-022-03889-y)
Supplement: Supplementary file 3 — Additional file 3. [file 12870_2022_3889_MOESM3_ESM.docx]

**Table S2. Primer sequences used in this study**

| **Primers for qRT-PCR** | |
| --- | --- |
| CsEF1α-F | AGACGATCTGGAAAGGAGATAGAG |
| CsEF1α-R  CsG_LG8.g33949.1-F | TCATGTCACGCACAGCAAAC  ATTCGCACAAGAAAGAACCAAC |
| CsG_LG8.g33949.1-R | CATAGTTATGCTGTTCCCATTGC |
| CsG_LG4.g53779.1-F | CTGAAAGATGTGAACAAAAAACTTAGAAC |
| CsG_LG4.g53779.1-R | TCACGAATGACATAGACAGCCTC |
| CsG_LG9.g33362.i1-F | GCATGCAAATAGAGCTCAGGC |
| CsG_LG9.g33362.i1-R | TCCTCCTCCAAAACTTGCTCA |
| CsG_LG7.g04334.i1-F | GCTTGATGCCAGGAAGTTCTG |
| CsG_LG7.g04334.i1-R | GGAGAGGAGTTTGGTCTTGGC |
| CsG_LG9.g43875.i1-F | AGCCAATGCTCAGTTTTACCAA |
| CsG_LG9.g43875.i1-R | TTCCCAAGCGATTCACCC |
| CsG_LG6.g50199.i1-F | TAAGGAAGAAGTTGGAGGAAAGTG |
| CsG_LG6.g50199.i1-R | CCGAGTGGGTAGGAAGAGGAT |
| CsG_LG7.g49236.1-F | TGAAAGGAATTACTATGGTGAGGAG |
| CsG_LG7.g49236.1-R | TGAGACAGTTGGTGTTGGGG |
| CsG_LG7.g11579.i1-F | CAGAGAAGCGCCAAAGAGTG |
| CsG_LG7.g11579.i1-R | CGAAGTGGATGTGAGGAAAATG |
| CsG_LG5.g64343.i1-F | TCAAAGTACAACAGCCGAATGC |
| CsG_LG5.g64343.i1-R | TGTGAGGAAAATGTGCCCAAT |
| MTCONS_00042147-F | CAAACCACCTGAGAAGAAACACA |
| MTCONS_00042147-R | TCAATCCAGAAAGATCAAGACCA |
| CsG_LG8.g58037.i1-F | ATACGGGATGATAGAGGATGGAG |
| CsG_LG8.g58037.i1-R | AAGATTGTTGGGATGGTCGG |
| CsG_LG6.g06424.i1-F | GCTGCCGTTTTCTGGAGG |
| CsG_LG6.g06424.i1-R | GTGGGGAAGGGATCTCCG |
| CsG_LG5.g56450.i1-F | TAGCCATCCAAAAGACCTCCG |
| CsG_LG5.g56450.i1-R | CGTAGTCTATGGACCCCACTGAC |
| CsG_LG3.g28582.1-F | TGGTTATGCTGTTGATGGTTGC |
| CsG_LG3.g28582.1-R | TCTATGGAAGTTACGGTGGCAG |
| LXLOC_026470-F | AGGCTGTAAAGCATTTCACTAACC |
| LXLOC_026470-R | GACAAATGAAATCAAACAATAAGGGT |
| LXLOC_037271-F | GTTAAGGAATGGGCTTTGGG |
| LXLOC_037271-R | AATGCACAAGGTGTGAATCAAAC |
| LXLOC_053379 -F | AAAGCCCAAAAGTTGTAGTCAGA |
| LXLOC_053379 -R | CCAATTCCAGAGAATCCTCCAA |
| LXLOC_086819-F | CGCCGCCTCCACCTTTTC |
| LXLOC_086819-R | CGACGGTCTGCCGCATAA |
|  | |
